# Personal experience: Hopes and fears - the road to recovery after psychotic illness

**DOI:** 10.1192/pb.bp.113.044024

**Published:** 2014-08

**Authors:** Aashish Tagore

In a previous article, I wrote a personal account of the stigmatising impact of an acute stress-induced psychotic episode in the context of being suspended from work following a false allegation. Here, I attempt to describe the psychological hurdles I’ve had to face in my recovery back to ‘full functioning’.

The psychotic episode took its toll on me. After the acute phase, I was constantly encouraged by both my treating psychiatrist and my care coordinator to take as much time as I could resting and recuperating. This made complete sense at face value: after all, the last thing any of us wanted was for me to feel unduly stressed and to experience a relapse. Despite this, my natural urge was to get back to work post haste. This is a strange trait that most of my medical colleagues will be able to relate to - for some reason we have an inherent sense of duty to our vocation, even if it is at the expense of our own health.

## Issues relating to the fear of relapse

### Recognising the signs and admitting to them

Although I was keen to return to work, I knew I had fears and anxieties. Of course, the main one was ‘what if I really do have some sort of relapse?’ I not only feared the reality of this experience, but wondered whether I would be able to recognise what it might look like - would I have the requisite insight and awareness to be able to detect early warning signs? What would these feel like? How would I be able to tease out the difference between slightly irrational thoughts in the context of the allegations I originally faced, and genuine paranoid ideation or delusional beliefs? Sometimes it can be a fine line. Would I find myself overreacting to every occasion when I felt a little bit anxious or vulnerable, or would I be able to contain it effectively? And even if I was able to recognise signs of a genuine relapse, would I really be able to effectively communicate these concerns to my consultant supervisor? What on earth would I say - ‘Erm, hi Dr X, I just thought I should let you know, I saw a patient gave me a sign to tell me they knew they needed to protect themselves from me. I’m not sure, maybe it’s just my mind playing tricks with me, just thought I’d let you know!’ You see, it’s one thing to have insight and awareness, it’s quite another to know what to do with it if and when things start to go wrong. I sometimes think I’d rather be completely oblivious to a relapse in such a situation, as this would then remove the need for me to seek help proactively. That’s one aspect I found most troublesome - would I really have the courage to tell someone, a work colleague, my supervisor, that I believed I was becoming ill again? Or, in reality, would I try to keep it to myself and hope it would pass with time, or I would be able to conceal it?

It must be so hard for patients with established illnesses that are relapsing in nature to actually seek help when they think they’re falling back into illness. After all, look at the potential repercussions - possible hospitalisation (for how long who knows), medication changes, and yet more shame and embarrassment for one’s loved ones. I definitely never, ever, want to go back to that horrid, dark place when my illness was at its peak, I simply don’t know if I’d be able to cope again. Aside from this, in the event of an emergent relapse, how would it manifest? Would I end up having a complete breakdown in the middle of a clinic? Would I embarrass or even scare a patient? Would my colleagues have to intervene? Would I have to be restrained? Maybe my mind was getting carried away, but now nothing was completely beyond the realms of possibility.

### Significance of experiencing another episode

Another aspect that frightens me is this: to have one psychotic episode within the context of what was a clearly extremely stressful period is one thing, but to have two or more psychotic episodes is something entirely different. Or at least this is how it would be viewed. As it stands, my psychiatrist is happy to label this illness as a transitory, fleeting one, a one-off in exceptional circumstances. But, were I to have a relapse, this would take me well and truly into the realms of what we psychiatrists like to call ‘severe and enduring mental illness’, a catch-all rubric used to describe conditions that follow a frequently relapsing and chronic course. This, of course, includes that most dreaded of diagnoses, paranoid schizophrenia. At the moment, I am happy to conceptualise what happened as stress-induced, because I can then assign external blame, in essence, that I was a helpless victim of circumstance. This way of thinking sits well with me, because it suggests I am not really vulnerable or susceptible to psychosis except in the most stressful of circumstances, where perhaps most people would have some kind of ‘nervous breakdown’. But to experience a relapse would change everything completely. Then, in my mind, I would be resigned to a lifetime of mental illness and the torture that accompanies it. I would undoubtedly feel depressed, resigned and hopeless for my future. I would suddenly be faced with an altogether different proposition and prognosis. And I doubt whether I could actually deal with that. How would I reconcile myself to the fact that this thing is probably going to happen time and again? To be honest, I’m not sure I could.

### Impact on medication decisions

The mere thought of experiencing a relapse is so fear-inducing for me that it has had an effect on my treatment decisions. Essentially, my treating psychiatrist felt that, as this was all ‘stress-related’, I should reduce my medication and discontinue it fairly quickly. Most patients with psychosis would love to be instructed by their psychiatrist to come off their medication, but to me, at that moment in time, it seemed crazy. I was fully aware that my antipsychotic medication had played no small part in my recovery, and in sustaining it. To stop it meant risking a relapse. And so I insisted that I would be staying on my medication ad infinitum, or at least, until I felt stable and safe enough in my mental health. And so I continue to take my prescribed risperidone daily. Indeed, on the odd occasion when I have missed a dose, I feel vulnerable. And so, it would seem, I have developed somewhat of a psychological dependency on my antipsychotics. And to be honest, I can’t see how I will ever be able to come off them: as I said before, I never, ever want to go back to that horrid, dark place again, and I will do everything to prevent that from happening.

It is not as if I want to be on this medication. There are many trade-offs for my peace of mind. I feel slowed down, mentally and physically, sometimes I feel so restless and can’t stop moving, and I feel emotionally blunted and flat. And worst of all, I’ve put on over two stone (12.7 kg) of weight in the course of 9 months. The increase in appetite is simply indescribable - it’s insatiable and then some. And of course, this has had a significant impact on my self-esteem and image, but that is the least of my worries. Recently, I have found out that my blood sugars are creeping up, and I meet criteria for impaired glucose tolerance. The threat of diabetes proper looms large. It’s almost as if I have to make a conscious decision between my mental health or my physical health. Do I risk another descent into madness, or do I risk all the diabetes-o-pathies? This is a serious consideration. For the time being, I have decided that I must remain mentally well, at whatever cost. In the meantime, I resolve to exercise a bit more.

## Return to work

With the help of my medication, and after 7 months away from work, I returned a couple of months ago. My return was on a phased basis, and I initially sat in on my consultant’s clinics. This compounded my sense of uselessness: there I was, an experienced specialist registrar, sitting and watching my consultant work through a busy clinic. But I knew it was in my best interests. Despite all my reservations and apprehension, I am pleased to report that things have gone pretty smoothly. Every day I come home without experiencing irrational or abnormal thoughts, I feel my confidence and self-belief returning, bit by bit, slowly but surely. I feel relieved, this thing could have derailed my career completely. Thankfully, I’m back doing what I love. My round trip from psychiatrist to psychiatric patient and back to psychiatrist is complete. My sense of purpose has returned, and I am enjoying my work. But I know, in the back of my mind, I will always have some degree of paranoia about relapsing. This fear will always remain with me. But for now, I feel quietly optimistic about my future well-being, my career and my life in general.

Also on a happier note, my wife is pregnant with our first child. This good news helped me get through the bleakest of times, and gave me the motivation to get better and move forwards from this. It gave me the reason to progress. However, even in this respect, the concerns relating to mental illness continue to dampen down our enthusiasm. Will our child inherit my apparent vulnerability to illness (and by that I mean psychosis) under stress, and will he suffer too? And if this did occur, how could I live with the sense of guilt? And so, despite my reasons to be cautiously optimistic, my psychotic experience will continue to haunt me in one way or another. An unforgettable experience, there is no doubting that. An unpredictable and uncertain future awaits me. Fingers crossed...

